# 

**Published:** 1853-07

**Authors:** 


### OUR DENTAL ABSTRACTS.

These are not as complete in this number as we intended. Dr. Hills article on Dental patents in the last number of the Journal, we intended to notice, also a translation of C. F. Delabarre's method of making continuous gum, from the News letter. Besides much other valuable and interesting matter, which we should have been glad to notice, but have not room in the present number. We hope in our next to continue these.

### PRESENT NUMBER OF THE REGISTER.

It will be perceived by our subscribers, that we have somewhat enlarged the Register. In the present issue we found this indispensable, and if our subscribers will remit us promptly their dues, and for the next volume, we think we shall be able to continue it the same size. This will add sixty-four pages to the size of the volume, making a volume in all, of over three hundred pages.

### SUBSCRIBERS.

There are many of our old subscribers who, we are sure merely neglect remitting their dues from year to year, until they really forget the amount of their indebtedness. We hope all such, on the receipt of this will attend to this matter. We now close the sixth volume, and have the satisfaction to know that our list of subscribers have gradually but constantly increased. We shall continue the work so long as health and circumstances permit.

### POSTAGE.

We have been pre-paying the Register to cash subscribers, paying in advance for Vol. 6. The labor and difficulty in making out every number a correct list is greater than the small amount which the postage amounts to would justify. And as we hope to give our subscribers an additional amount of reading matter we shall discontinue this.

---

### MR. JOHN D. CHEVALIER.

We draw attention to the removal of this gentleman's Dental furnishing establishment. which is now 360 instead of 193 Broadway, New York. Mr. Chevalier is known as one of the most extensive Dental Instrument manufacturers in the United States. More or less of his instruments are found in almost every Dental office, and their superior quality generally acknowledged We are satisfied the Profession will soon find Mr. Chevalier's whereabouts, and as his instruments are No, 1, they will sell just as well at No. 360 as at 193. So that his bonus of $7000 for removal will be clear gain.

### STOCKHOLDERS OF THE OHIO COLLEGE OF DENTAL SURGERY.

Will please bear in mind that the last instalment on their stock is due first of August next. And it is desired that the payments should all be promptly met as heretofore, for the Trustees appointed at our last meeting, wish to get the deed made out, and hav every thing arranged, so that certificates of stock may be issued in due form by our next meeting.

---

### MEDICAL AND DENTAL JOURNALS RECEIVED.

American Journal of Dental Scien e.
Dental Recorder.
Dental News Letter.
Semi Annual Dental Fxpositor.
St. Louis Medical and Surgical Journal.
Ohio Medical and Surgical Journal.
Western Lancet.
Boston Medical and Surgical Journal.
Charleston Medical Journal and Review.
Medical Chrcnicle, or Montreal Monthly Journal of Medicine and Surgery.

Received,—Respiration subservient to nutrition. A Thesis, by E. LEIGH M. D. Townsend, Mass. The subject is presented in a very clear and lucid manner, and gives much valuable information. We thank the author or some friend for the pamphlet.

### NINTH ANNUAL ANNOUNCEMENT
OF
### THE OHIO COLLEGE OF DENTAL SURGERY.
**Session of 1853–'54.**

The regular course of lectures in this institution, commences the first Monday of November, and closes on the third Wednesday of February.

**FACULTY.**

JAMES TAYLOR, M. D., D. D. S.
*Professor of the Principles and Practice of Dental Surgery.*

THOMAS WOOD, M. D.,
*Professor of Anatomy and Physiology.*

J. B. SMITH, M. D.,
*Professor of Pathology and Therapeutics.*

JOHN ALLEN, D. D. S.,
*Professor of Operative Dentistry.*

H. R. SMITH, D. D. S.
*Professor of Mechanical Dentistry.*

G. WATT, M. D.,
*Lecturer on Chemistry,*

**EXAMINING COMMITTEE.**

DENTAL. A. S. TALBERT, D. D. S., of Lexington, Ky.
W. H. GODDARD, D. D. S., of Louisville, Ky.
H. E. PEEBLES, D. D. S., of Lexington, Mo.
MEDICAL. PROF. MENDENHALL, M. D., of Cincinnati, O.
ISRAEL DODGE, M. D., of Cincinnati, O.

The Faculty in issuing this their Ninth Annual Announcement, take pleasure in stating to the Profession that the Institution is based upon a firm foundation, being chartered by the state and owned and governed by stockholders (most of whom are regular Dental Practitioners) in connection with a board of Trustees. In the reorganization of the Faculty, the Profession have every assurance that the interest of the school has been consulted and that the course of instruction will be such as to meet the wants of the dental student.

A building suitable in all the conveniences for a Dental College is owned by the Profession and dedicated to the advancement of dental science.

The library is increasing in useful and appropriate books, and a fund is secured for the continued increase of this and such apparatus as may be needed.

The Professor of Mechanical Dentistry has made arrangements to open the laboratory and infirmary, by the middle of October. Every facility will be afforded the student, and every thing requisite furnished, but such materials as are of a perishable nature.

Tickets for the course, $100,00 ; Matriculating Ticket, 5,00 ; Diploma Fee, $25,00. Good board can be had at from $2,50 to $3,00 per week.

JAMES TAYLOR, *Dean.*

### RECEIPTS FOR THE REGISTER.

J. P. Ulrey, Rising Sun, Ia., vol. 5,....$2
J. A. Dougherty, Bowling Green, Ky., vols. 5 and 6,.................. 4
J. L. Milton, Lexington, Miss. vols. 4 & 5, 3
J. M. Abrams, Brownsville, Pa., vol. 6, 2
J. F. Wilson, Greencastle, Ia., vol. 6... 2
Nathan P. Allen, Gascon, Ky., vol. 6... 2
W. S. Jones, Russelsville, Ala., vol. 6,. 2
H. B. Sisson, Fairfield, Iowa., vol. 6,.. 2
Saul P. Cutter, Holley Spring, Miss., vol. 6,.................. 2
D. J. Patton, Brownsville, Pa., vol. 6,. 2
W. G. Redman, Henderson, Ky., vol. 6, 2
B. A. Kennidy, Wilmington, N.C., vol. 6, 2
Hiram A. Hartzell, Addison, Pa., vol. 6, 2
H. Baker, Cold Water, Mich., vol. 6,... 2
C. F. Munter, Bolivia, Tenn., vol. 6,... 1
David Parkineon, Independence, Pa., vol. 6,.................. 2
J. E Hewlett, Saint Josephs, Mo., vol. 6, 2
J. L. Walker, Hopkinsville, Ky., vol. 6 2
John Hood, Greensbury, Ia., vol. 6,... 2
S. P. Miller, Worcester, Mass., vol. 6,.. 2
C. R. Thomas, Roxbury, Mass., vol. 6,.. 2
J. T. Freeman, Boston Mass., vol., 6,... 2
T. A. Lynn, Lexington, Ia. vol. 6,...... 2
G. G. Samuel, St. Louis, Mo., vol. 6,... 2
S. Fleager, Vincennes, Ia., vol. 6,...... 2
J. Hough, Lagrange, Texas, vol. 6,...... 2
A. W. French, Springfield, Ia., vol. 6,.. 2
James A. Tenney, Marietta, O., vol. 6. 2
Hiram A. McDaniel, Hopkinsville, Ky, vol. 5,.................. 2
Wm. Grimes, Dubuque, Iowa, vol. 6,.. 2

### DENTAL
### FURNISHING ESTABLISHMENT,

**Corner of Fourth and Walnut streets, Cincinnati, O.**

The Subscriber keeps constantly on hand, at Wholesale and Retail, every article needed in the practice of the

### DENTAL PROFESSION.

His stock is part composed of the following, viz:

PORCELAIN TEETH, Stockton's Manufacture,
Do Do Jones, White & Co's. do
Do Do Alcock's. do
GOLD FOIL, Abbey's, Ashmead's Leslie's and Jones',
FORCEPS of the latest and most approved patterns,
GOLD AND SILVER MOUNTED PEARL and
IVORY HANDLED INSTUMENTS.

**A Superior Article of Gold Plate.**

Cut to pattern at ONE DOLLAR a dwt.

ALSO—GOLD SPRINGS alloyed with Platina—a late improvement.

J. M. BROWN.

WE, Members of the Dental Profession in the city of Cincinnati, and *Patrons* of the above establishment, do most cheerfully recommend the same to the confidence and patronage of our brethren in the Mississippi Valley,

H. CRANE.
JAMES TAYLOR,
EDWARD TAYLOR,
CHARLES BONSALL,
W. E. IDE,
JESSE W. COOK,
M. RODGERS,
JOHN ALLEN,
WM. M. HUNTER,
ROSS & BERRY.

---

### POST MASTERS,

Will please send back the Register when it is not taken out of their office. The name of the individual to whom it is addressed, and name of post office should be plainly written on the envelope.

Exchanges, please direct, "Dental Register, Cincinnati, Ohio."

